# Chronic Pain: An Update of Clinical Practices and Advances in Chronic Pain Management

**DOI:** 10.5152/eurasianjmed.2022.22307

**Published:** 2022-12-01

**Authors:** Selin Guven Kose, Halil Cihan Kose, Feyza Celikel, Serkan Tulgar, Alessandro De Cassai, Omer Taylan Akkaya, Nadia Hernandez

**Affiliations:** 1 Department of Pain Medicine, Health Science University Derince Training and Research Hospital, Kocaeli, Turkey; 2 Department of Physical Therapy and Rehabilitation, Sakarya Training and Research Hospital, Sakarya, Turkey; 3 Department of Anesthesiology and Reanimation, Samsun University Faculty of Medicine, Samsun Training and Research Hospital, Samsun, Turkey; 4 Section of Anesthesiology and Intensive Care, University Hospital of Padova, Padova, Italy; 5 Department of Pain Medicine, Health Science University Ankara Etlik City Hospital, Ankara, Turkey; 6 Department of Anesthesiology, Memorial Hermann Hospital, Texas Medical Centre, Houston, USA.

**Keywords:** Chronic pain, pain medicine, ultrasound, interventional, nerve block, pain management

## Abstract

Chronic pain affects a significant amount of the population and represents a heavy personal and socioeconomic burden. Chronic pain mechanisms can be categorized as nociceptive, neuropathic, or nociplastic. Although mechanism-based pain treatment is optimal, different types of pain mechanisms may overlap in patients. Recently, the biopsychosocial model with the multidisciplinary pain management program is widely accepted as one of the most effective methods to assess and manage chronic pain. The treatment of chronic pain consists of a personalized, stepwise, and multimodal approach that includes pharmacotherapy, psychotherapy, integrative treatments, and interventional procedures. Somatic and peripheral nerve blocks for the treatment of chronic pain are often deferred. With the increasing use of ultrasound in pain medicine, newly defined interfascial plane blocks, which may be performed alone or as an adjuvant to multimodal management, have gained popularity. Adequate pain management can improve physical functioning, mental health and quality of life indicators, and reduce pain chronification. The aim of this current article is to perform a comprehensive and updated review of existing treatment options, particularly interfascial plane blocks in chronic pain syndromes.

**Main Points**

- Chronic pain affects a significant amount of the population and represents a heavy personal and socioeconomic burden.
- Ultrasound-guided interfascial plane blocks are feasible and safe techniques that can be performed alone or in combination with multimodal chronic pain management.
- Although mechanism-based pain treatment is optimal, several mechanisms converge under the umbrella of chronic pain and nociceptive, neuropathic, or nociplastic pain may overlap in patients.
- Adequate pain management can improve physical functioning, mental health, and quality of life indicators and reduce pain chronification.

## Introduction

Pain is defined as an unpleasant sensory and emotional experience associated with, or resembling that associated with, actual or potential tissue damage.^1^ Chronic persistent pain with an affective-arousal component often seriously affects the patients’ quality of life, daily living activities, psychological status, and overall well-being.^2,3^ Adequate pain evaluation is key to the management of chronic pain and requires careful patient history, evaluation of red flags, optimized physical examination and signs, management of psychosocial problems, and performance of specialized tests.

### Classification of Chronic Pain

Chronic pain is classified as primary, that is, unrelated to other medical conditions and independent of other pain generators, or secondary, that is, due to other underlying conditions encompassing cancer pain, chronic postoperative pain, neuropathic pain, musculoskeletal pain, and visceral pain.^4^ Additionally, chronic pain is classified into nociceptive, neuropathic, and nociplastic pain. Nociceptive pain results from activation of nociceptors secondary to actual or imminent damage to non-neural structures. Neuropathic pain is defined as pain caused by pathology or damage affecting the somatosensory nervous system. It is typically accompanied by sensory abnormalities, such as allodynia and numbness, more prominent pain paroxysms, and, depending on the nerves affected, neurological findings. Nociplastic pain results from altered processing of pain signals despite no clear evidence of damage or pathology of the somatosensory system. In clinical practice, these subclassifications often overlap.^4,5^ For example, patients with cancer and spinal pain may have a mixed pain phenotype.

### Clinical Practices in Chronic Pain Syndromes

Chronic pain results from a combination of many biological, psychological, and social factors.^6,7^ Although mechanism-based pain treatment is optimal, several mechanisms converge under the umbrella of chronic pain, and defining the mechanisms behind pain can be challenging in clinical practice. Hence, the treatment of chronic pain consists of a stepwise approach, which should be tailored to the presenting patient, and implemented in a multimodal manner.

Clinical guidelines recommend starting with conservative treatments such as physical therapy, exercises, and/or psychologically based interventions, including commitment and acceptance therapy and cognitive behavioral therapy.^8,9^ Several types of non-opioid drugs are used to treat both neuropathic and non-neuropathic pain. Topical and oral nonsteroidal anti-inflammatory drugs (NSAID) have proven their efficacy in non-neuropathic pain and are considered as first-line treatment for osteoarthritis and other inflammatory chronic diseases, and short-term oral NSAIDs are commonly prescribed for the treatment of low back pain.^10,11^ Among the antiepileptic drugs, gabapentin and pregabalin have been used successfully in chronic neuropathic pain conditions, including post-herpetic neuralgia, diabetic peripheral neuropathy, and spinal cord injury. Antidepressant drug classes, particularly tricyclic antidepressants and serotonin norepinephrine reuptake inhibitors, are indicated for neuropathic pain.^12^

Although opioids are the standard pharmacological agent used for acute pain, long-term use of opioids is not considered a first-line treatment for the treatment of any form of chronic pain. The overall mechanism of action of opioids is inhibition of the ascending excitatory pathway and activation of the descending inhibitory pathway. In addition, opioids provide analgesic effects through the activation of peripheral opioid receptors. Long-term opioids can become a problem rather than a solution in chronic pain, with the disadvantages being their effect on the endocrine and immunological system and opioid-induced hyperalgesia.^13,14^ Intravenous infusions of various pharmacological agents such as lidocaine have been used for pain relief.^15^

### Clinical and Research Consequences

#### Non-Surgical Interventional Treatment

When oral medication to control symptoms is not effective, minimal invasive interventional pain techniques may be considered for the treatment of chronic pain (Table 1). The right selection of patient characteristics and minimally invasive procedures could contribute to greater success of interventions for pain relief in clinical practice. The ideal patients for interventions are those with pathology concerning dermatomal pain distribution patterns and without depression, opioid use, high baseline disability, and pain scores.^16^

The use of epidural steroid injections including transforaminal, interlaminar, and caudal and fascial blocks in patients with lumbar radicular pain are effective examples of multimodal interventional treatments.^17^ Radiofrequency (RF) treatment is a common treatment technique for cervical and lumbar facet joint and sacroiliac joint pain and knee osteoarthritis with long-term pain relief.^18^ In general, there are 2 types of RF procedures. In the first type, continuous RF has provided a satisfactory method, such as thermocoagulation of the medial branch. The second type is pulsed radiofrequency (PRF) treatment for peripheral neuropathies, painful trigger points, and application of the caudal epidural and dorsal root ganglion in patients with neuropathy or radiculopathy. The use of PRF is said to be encouraged by a significant reduction in complications or side effects, even though it is as successful as thermal or conventional RF in this area.^19^ Neuromodulation technology including spinal cord stimulation and peripheral nerve stimulation is an expanding area of chronic pain treatment.^20^

With the increasing use of ultrasound in pain medicine, newly defined interfascial plane blocks have gained popularity.^21,22^ Furthermore, new knowledge about the anatomical content of the fascia and its involvement in the etiopathogenesis of chronic pain syndromes has enhanced the utility of interfascial plane blocks in chronic pain management.^23-25^ There is growing evidence in the literature that interfascial plane blocks with local anesthetics and corticosteroids are offered for therapeutic purposes to patients with chronic pain.^26,27^

Recently, the indications have been expanded by applying it to manage chronic pain conditions such as low back pain and myofascial pain syndrome (MPS) in the neck and upper back.^28,29^ Furthermore, interfascial block catheter procedures can provide long-term analgesia with noticeable advantages of safety and sono-anatomic simplicity.^30^

#### Chronic Pain Syndromes

Myofascial pain syndrome is a pain that originates in the muscles and surrounding fascia and is accompanied by sensory and motor symptoms as well as autonomic phenomena. It is characterized by the presence of myofascial trigger points.^31^ Currently, a variety of superficial injection techniques are commonly used in MPS, including trigger point injections, local anesthetic injections, and dry needling.^32^ Recently, attention has focused on the fascia as a possible cause of MPS. In MPS, the erector spinae plane block and the rhomboid intercostal block are the most commonly performed plane blocks for pain relief.^33^ Furthermore, different kinds of interfascial plane blocks have been successfully performed in chronic pain syndromes such as acute herpes zoster.^34,35^ Pain lasting for more than 3 months following a herpes zoster infection in one or more sensory dermatomal distributions along with skin changes is called post-herpetic neuralgia. Multidisciplinary team management of severe post-herpetic neuralgia with a combination of pharmacological, non-pharmacological, and interventional approaches is the key to optimize pain management.^36-39^

Headaches can be primary and secondary. Diagnosing the type of headache is the most important step in the management and success of treatment. Currently, the International Headache Society International Classification of Headache Disorders is the widely used classification for headache. Red flags including sudden severe headache, headache with neurological deficit and/or seizures, headache associated with a new onset of headache in a 50-year-old and older, change in character or pattern of headaches, and cancer or immunosuppression history have to be ruled out. Clinical guidelines and research recommend abortive and preventive strategies.^40^

Osteoarthritis is the most common joint disorder and the most common cause of knee and hip pain worldwide.^41^ Challenges remain in the management of osteoarthritis to slow progression and reduce the number of major surgeries. Therefore, strategies are being developed to enable patients with severe pain and functional limitations to improve quality of life.^42,43^ Keeping active is the key to treating osteoarthritis. Intraarticular injections and genicular nerve blocks have a role to play in osteoarthritis.^44^

Lumbar radicular pain characterized by a radiating pain in one or more lumbar dermatomes, has become one of the biggest problems and the leading cause of disability worldwide.^44^ The structures, including vertebrae, facet joints, intervertebral discs, and neurovascular elements that make up the lumbar spine, tend to be affected by various stressor factors, and these structures can cause lumbar radicular pain alone or conjointly. It is most commonly associated with lumbar spinal stenosis, herniated intervertebral disc, and failed back surgery syndrome. The primary goal of treating lumbar radicular pain is to improve physical functionality and enhance the patient’s quality of life. Non-pharmacological, non-invasive treatments such as exercise therapy and injection techniques are commonly performed to treat low back pain.^44,45^ Recently, a broad range of interfascial planes blocks are being used to treat chronic pain conditions such as lumbar and shoulder pain, and these may be a good alternative for the management of chronic low back pain in the future.^17,28,29,43^

Chronic post-surgical pain is defined as chronic pain that develops after a surgical procedure or tissue injury lasting at least 3 months after surgery. Thoracotomy, sternotomy, mastectomy, cholecystectomy, and inguinal hernia surgery are the surgical procedures known to lead to chronic post-surgical pain with a high incidence. Peripheral and central sensitization are considered pathophysiological mechanisms for the development of chronic post-surgical pain.^46^ Preventive strategies are the main key to reduce the incidence of chronic pain after surgery; hence multimodal analgesia with regional blocks during the perioperative period should be considered.^47-49^

Chronic pelvic pain is associated with pelvic structures encompassing gastrointestinal, genitourinary, pelvic floor musculoskeletal, and neural tissues. The source of pelvic pain can be visceral, somatic, neuropathic or mixed, and pelvic pain has significant psychosocial consequences. The persistence of persistent chronic visceral pain is explained by central sensitization and neuroplasticity. Pelvic floor exercises, relaxation techniques, complementary therapy, and psychological interventions are all part of a multidisciplinary approach. Peripheral nerve injections, including pudendal nerve, ilio-inguinal, ilio-hypogastric, and genitofemoral blocks, are performed to inhibit ascending inputs. Besides neuromodulation strategies, the ganglion of impar and the superior hypogastric plexus are the main targets.^50,51^

The etiopathogenesis of cancer-related pain is often multifactorial and has nociceptive, neuropathic, and visceral components. A direct result of the disease (tumor invasion, compression of neuronal structures bone metastasis, and ischemic necrosis), oncologic therapies (chemotherapy- and radiotherapy-related neuropathic pain and chronic post-surgical pain), and complications of critical illness (chronic illness neuropathy and myopathy) are all known to cause cancer pain.^52^ A multidisciplinary pharmacological and non-pharmacological and intervention approach and psychological support should be implemented. Knowledge of neuro-anatomy and peripheral and central nerve block techniques can be used in cancer-related pain. Somatic and peripheral nerve blocks have proven effective for malignant pain control even early in the disease course.^53^ Destructive neurosurgical procedures are not included in the 3-step World Health Organization pain ladder and should be offered to patients who are refractory to opioid therapy and other medical and interventional procedures.^54^

## Conclusion

Finally, chronic pain is a major public health problem worldwide affecting the patients’ quality of life, functional status, and psychological status. This review aims to provide a clear summary of evidence-based best practice in light of the significant prevalence of chronic pain and the wealth of information on treatment options. This will potentially lead to better treatment options and better long-term outcomes if there is a greater emphasis on comprehending chronic pain.

## Figures and Tables

**Table 1.** Block Techniques for Chronic Pain

Region	Block	Volume* (mL)
Head	Gasserian ganglion block	2-4
	Occipital nerve block	1-4
	Maxillary and mandibular nerve block	4-6
Cervical	Cervical nerve root block	1-2
	Cervical plexus block	9-15
	Facet joint injection	0.25-0.5
	Medial branch block	0.5-1
Thoracolumbar	Paravertebral block	2-5
	Erector spinae plane block	10-20
	Rhomboid intercostal block	10-15
	Serratus plane block	10-30
	Thoracolumbar interfascial plane block	20
	Intercostal nerve block	2-4
	Facet joint injection	0.25-0.5
	Medial branch block	0.5-1
Anterior abdominal wall	Transversus abdominis plane block	10-20
	İlioinguinal, iliohypogastric, and genitofemoral nerve block	10-20
		8-10
Lower extremity	Femoral nerve block	15-20
	Sciatic nerve block	20-25
	Pericapsular nerve block	15-20
Autonomic nerve blocks	Stellate ganglion block	4-6
	Lumbar sympathetic block	8-10
	Celiac plexus block	8-10
	Superior/inferior hypogastric plexus block	8-10
	Impar ganglion block	4-5

^*^Local anesthetic (0.25% bupivacaine) volume used in blocks per side as single injection. This table is for informational purposes only based on authors’ clinical practice. For the use of other local anesthetics, such as lidocaine and ropivacaine, it is recommended to consult the literature. About 4-8 mg dexamethasone and/or 1:200 000 epinephrine could be added as an adjuvant drug.
